# Absence of evidence is not evidence of absence: Nanopore sequencing and complete assembly of the European lobster (*Homarus gammarus*) mitogenome uncovers the missing *nad2* and a new major gene cluster duplication

**DOI:** 10.1186/s12864-019-5704-3

**Published:** 2019-05-03

**Authors:** Han Ming Gan, Frederic Grandjean, Tom L. Jenkins, Christopher Mervyn Austin

**Affiliations:** 1 Deakin Genomics Centre, Deakin University, Geelong, VIC 3220 Australia; 2 Centre for Integrative Ecology, School of Life and Environmental Sciences, Deakin University, Geelong, VIC 3220 Australia; 3 School of Science, Monash University Malaysia, Bandar Sunway, 47500 Petaling Jaya, Malaysia; 4 Monash University Malaysia Genomics Facility, Monash University, Bandar Sunway, 47500 Petaling Jaya, Malaysia; 5 Laboratoire Ecologie et Biologie des Interactions, UMR CNRS 7267 Equipe Ecologie Evolution Symbiose 5 rue Albert Turpin, 86073 Poitiers, Cedex, France; 6 Department of Biosciences, College of Life and Environmental Sciences, University of Exeter, Exeter, UK

**Keywords:** *Homarus gammarus*, Mitogenome, Nanopore sequencing, Lobster, Tandem duplication, PCR bias

## Abstract

**Background:**

The recently published complete mitogenome of the European lobster (*Homarus gammarus*) that was generated using long-range PCR exhibits unusual gene composition (missing *nad2*) and gene rearrangements among decapod crustaceans with strong implications in crustacean phylogenetics. Such atypical mitochondrial features will benefit greatly from validation with emerging long read sequencing technologies such as Oxford Nanopore that can more accurately identify structural variation.

**Results:**

We re-sequenced the *H. gammarus* mitogenome on an Oxford Nanopore Minion flowcell and performed a long-read only assembly, generating a complete mitogenome assembly for *H. gammarus*. In contrast to previous reporting, we found an intact mitochondrial *nad2* gene in the *H. gammarus* mitogenome and showed that its gene organization is broadly similar to that of the American lobster (*H. americanus*) except for the presence of a large tandemly duplicated region with evidence of pseudogenization in one of each duplicated protein-coding genes.

**Conclusions:**

Using the European lobster as an example, we demonstrate the value of Oxford Nanopore long read technology in resolving problematic mitogenome assemblies. The increasing accessibility of Oxford Nanopore technology will make it an attractive and useful tool for evolutionary biologists to verify new and existing unusual mitochondrial gene rearrangements recovered using first and second generation sequencing technologies, particularly those used to make phylogenetic inferences of evolutionary scenarios.

**Electronic supplementary material:**

The online version of this article (10.1186/s12864-019-5704-3) contains supplementary material, which is available to authorized users.

## Background

The European lobster (*Homarus gammarus* (Linnaeus, 1758)) is an economically important crustacean species with a broad geographic range across the eastern Atlantic Ocean, extending from the Arctic Circle to Morocco [1].The only other species placed in the genus *Homarus* is the closely related American lobster (*Homarus americanus* (Fabricius, 1775)) with natural range on the opposite side of the North Atlantic [2]. Despite its socio-economic importance, early population genetics of the clawed lobsters employing various genetic markers such as mtDNA, microsatellites and random amplified polymorphic DNA focused on the American lobster. It is only in the last two decades that the genetic diversity and population structure of *H. gammarus* have been actively investigated using molecular genetic techniques [3–6]. One of the most comprehensive genetic investigations of *Homarus gammarus* was by Triantafyllidis and co-workers who sampled 3283 individuals from 44 populations across its native geographical distribution and analyzed the diversity and distribution of restriction fragment length polymorphism of a 3-kb mitochondrial DNA amplicon [7].

The complete mitogenome of *H. americanus* has been sequenced, assembled and annotated and is consistent with the pancrustacean gene order [8]. However, in stark contradistinction, the recently published *H. gammarus* mitogenome [9] deviates substantially as it was reported to have not only a large inversion but to be also missing the *nad2* genes and a few tRNA genes [10]. While minor and major departures from the pancrustacean gene order have been observed in multiple lineages of decapod crustaceans notably among scampi and Southern Hemisphere freshwater crayfish species [11–16], all 13 mitochondrial protein-coding genes are always present in the assembled mitogenome occasionally with some species having duplicated tRNA genes and occassionally the control region [14]. The finding of mitogenome structural variations is of interest as molecular synapomorphies and for providing insights in mitogenome evolution and it is quite rare to find such significant mitogenome rearrangements and missing genes between closely related species. A necessary caveat, however, is the assumption that mitogenome studies reporting rearrangements and missing genes have used appropriate wet lab and bioinformatics protocols that are robust to potential errors or misinterpretations. It is thus always beneficial that such atypical findings be verified by other laboratories.

Traditionally, complete mitogenomes have been recovered using long-range PCR followed by multiple Sanger sequencing reactions (primer walking) using overlapping fragments to produce a complete assembly. One of the assumptions of this approach is that the mitogenome of interest has a gene order that is identical to, or at least highly similar to the reference sequence. In other words, species with mitogenomes with substantial gene rearrangement relative to an appropriate reference mitogenome (usually a related species) will present a significant challenge to the complete recovery of mitogenomes using PCR-based approach as this will generally lead to failed PCR reactions, amplicon of unanticipated lengths and multiple amplicons as well as the potential amplification of nuclear-derived mtDNAs [17–19]. The advent of high-throughput short-read sequencing technologies has greatly enhanced the accuracy and efficiency of mitogenome recovery by allowing mitogenomes to be sequenced without prior sequence knowledge or gene order [20, 21]. By sequencing multiple small segments of the DNA (~ 100–250 bp) in parallel, such an approach essentially removes the assumptions and limitations of long-range PCR and also allows the recovery of mitogenomes from degraded DNA samples including those from museum specimens [22, 23]. Nevertheless, a major pitfall of this technology is the inability of short reads to span highly repetitive regions that exist in some mitogenomes particularly in the control region. This means that assemblies may give a fragmented mitogenome that precludes the accurate inference of gene order organization, although gap closing using custom design primers and Sanger sequencing can be successful in resolving such incomplete assemblies [24].

Three common approaches are used for the assembly of mitogenome from short read data e.g. whole mitogenome reference mapping, bait-and-extend from a single gene and de novo assembly; yet, these approaches can lead to different outcomes when applied to the same dataset [25]. Whole mitogenome reference mapping usually works well when the sequences are obtained from individuals from the same or a closely related species. However, some caution is advised in assembling mitogenomes in this way as even congeneric species can show variations in mitochondrial gene order as recently reported in the Southern Hemisphere burrowing freshwater crayfish, *Engaeus* spp. [14]. Inferring novel mitochondrial gene organization is generally more robust using either de novo assembly or single gene baiting approach as they do not make assumptions on a prior gene order information. Failure to recover a complete circular mitogenome is usually associated with anomalies in the sequence composition ranging from an overly repetitive control region to large-scale tandem duplications that will require gap-closing via PCR which is not always successful.

The recent improvements in Oxford Nanopore technology most notably in its read accuracy, flow cell stability, and sequencing algorithm have truly democratized long-read sequencing, providing individual researchers with sequencing capacity that is comparable to those previously found only in large sequencing centers [26]. To date, several studies have explored combining accurate Illumina reads with long (> 1 kb) but more error-prone Nanopore reads to improve whole genome assemblies [27–29]. More recently, this approach has been used to assemble the complete mitogenome (~ 16 kb) of the economically important green-lipped mussel [30]. However, the mitogenome exhibits the typical 13 protein-coding genes, 22 tRNAs, 2 rRNAs and a control region, suggesting that Illumina short read data alone can also accomplish the same objective as reported in various mussel mitogenomic studies [31–34]. Unlike the green-lipped mussel mitogenome, the *Homarus gammarus* mitogenome represents an interesting case due to its atypical gene organization that has been partially verified using PCR across a few individuals in a follow-up study [10]. Furthermore, the high genetic variation observed in the 3 kb mitochondrial region that excludes the control region is unexpected [7] but can be explained by the presence of a large tandem duplication event followed by pseudogenization which warrants further investigation. In this study, we generated the first Illumina dataset for *Homarus gammarus* providing strong evidence for structural incongruence with the published *H. gammarus* mitogenome. By fully exploiting the ability of Nanopore long-read to span large repeats, we were able to circularize the mitogenome leading to the discovery of the large tandemly duplicated mitogenome region with strong evidence of pseudogenization and the presence of the purportedly missing *nad2* gene.

## Methods

### Sample collection and gDNA extraction

A wild-caught European lobster from the Atlantic Ocean was purchased from a commercial fisherman. Approximately 500 mg of tail muscle tissue was dissected and preserved in 95% ethanol. DNA extraction was performed using an SDS- based lysis method followed by chloroform purification as previously described [35]. To confirm mitogenome features, notably the presence of *nad2* gene, additional European lobsters were collected from five different and distant European regions (Fig. 2c) as previously described [36].

### Illumina sequencing

One hundred ng of gDNA was sonicated to a fragment size of 500 bp using a Covaris ultrasonicator and processed using NEB Ultra DNA library prep kit according to the manufacturer’s instructions. The constructed library was quantified and size-estimated using Qubit and Tapestation2100, respectively. Sequencing of the library was performed on an Illumina MiSeq located at the Monash University Malaysia Genomics Facility using the run configuration 2 × 250 bp.

### Nanopore sequencing

Two μg of gDNA was repaired using NEB FFPE Repair Mix and subsequently processed using the Nanopore LSK108 kit according to the manufacturer’s instructions. The library (premixed with loading beads) was pipetted into a previously used and washed FLO-MIN106 flow cell. The flow cell was fixed to a MINION device and sequencing was performed for 16 h (overnight) on a Linux Ubuntu desktop through the MinKnow software version 2.0.

### Mitogenome assembly and annotation

Illumina-only mitogenome assembly was performed using two reference-based assemblers (MITObim and NOVOplasty) and a de novo assembler (Megahit) [37–39]. For MITObim, 3 separate assemblies were performed using different mitochondrial gene baits (*nad2*, 16S rRNA, and *cytb*) from the American Lobster, *H. americanus* (GenBank accession number: NC_015607). Completeness and the presence of anomalies in the assembled mitogenome were determined by conducting BLASTX search of the contig against the 13 mitochondrial proteins from *H. americanus*. For long-read only assembly, Nanopore long-reads were first aligned to the mitogenomes of *H. americanus* and *H. gammarus*. The aligned reads were error-corrected, trimmed and assembled using CANU v1.7 [40]. The graphical fragment assembly of contig flagged as circular by CANU was visualized on Bandage v0.8.1 [41]. A self-against-self nucleotide similarity search of the assembled contig was performed and visualized with EasyFig v2.2.2 [42] to confirm the presence of highly similar flanking regions in the contig. Polishing of the CANU assembly used Unicycler_Polish module in Unicycler v0.4.4 that mapped Illumina reads to the raw assembly using Bowtie2 (−-very-sensitive-local) and piped the alignment file into Pilon v1.22 to specifically correct for small indel and SNP [43–45]. Circularization of the polished mitogenome and re-orientation of the contig 5′ end to *cox1* gene used Circules.py python script that is part of the MITObim v1.9 software [39]. The circularized mitogenome was polished again to fix any remaining assembly errors in sequence previously originating from the flanking regions of the uncircularized contig. These errors may have been missed in the first polishing step due to the repetitiveness and lower read coverage in the flanking regions. The polished mitogenome was annotated using the MITOS webserver [46] and each protein-coding gene was manually inspected with their start and stop position adjusted when necessary using the *H. americanus* mitogenome as the reference. The polished *H. gammarus* mitogenome was subsequently used as the reference to reconstruct the mitogenomes of the 5 additional *H. gammarus* individuals through MITObim v1.9 “—quick” option.

### Visualization of mitochondrial gene organization and alignment

Nad2 proteins were aligned and visualized with MAFFT v7.123b and ESPript v3.0, respectively [47, 48]. Maximum likelihood tree based on the amino alignment was inferred using IQ-TREE v1.65 with 1000 ultra-fast bootstrap replicates [49]. Circular comparisons between the newly assembled *H. gammarus* mitogenomes used BRIG v0.95 [50] while linear comparison of gene organization between *Homarus* spp. used EasyFig v2.2.2 [42]. Alignment of Illumina and Nanopore reads to the complete mitogenome was performed using bowtie2 v2.3.0 and minimap2 v2.10-r761, respectively and visualized in IGV v2.4.5 [51, 52]. GC plot of the mitogenome (windowsize = 500 bp) was constructed in Artemis v17.0.1 [53]. Nucleotide and amino acid alignment of pseudogenes used MACSE v2.01 (gc_code =5) followed by alignment visualization in Jalview [54, 55].

## Results

### The mitochondrial NADH dehydrogenase subunit 2 (*nad2*) gene has not been lost in *Homarus gammarus*

**Fig. 1:**
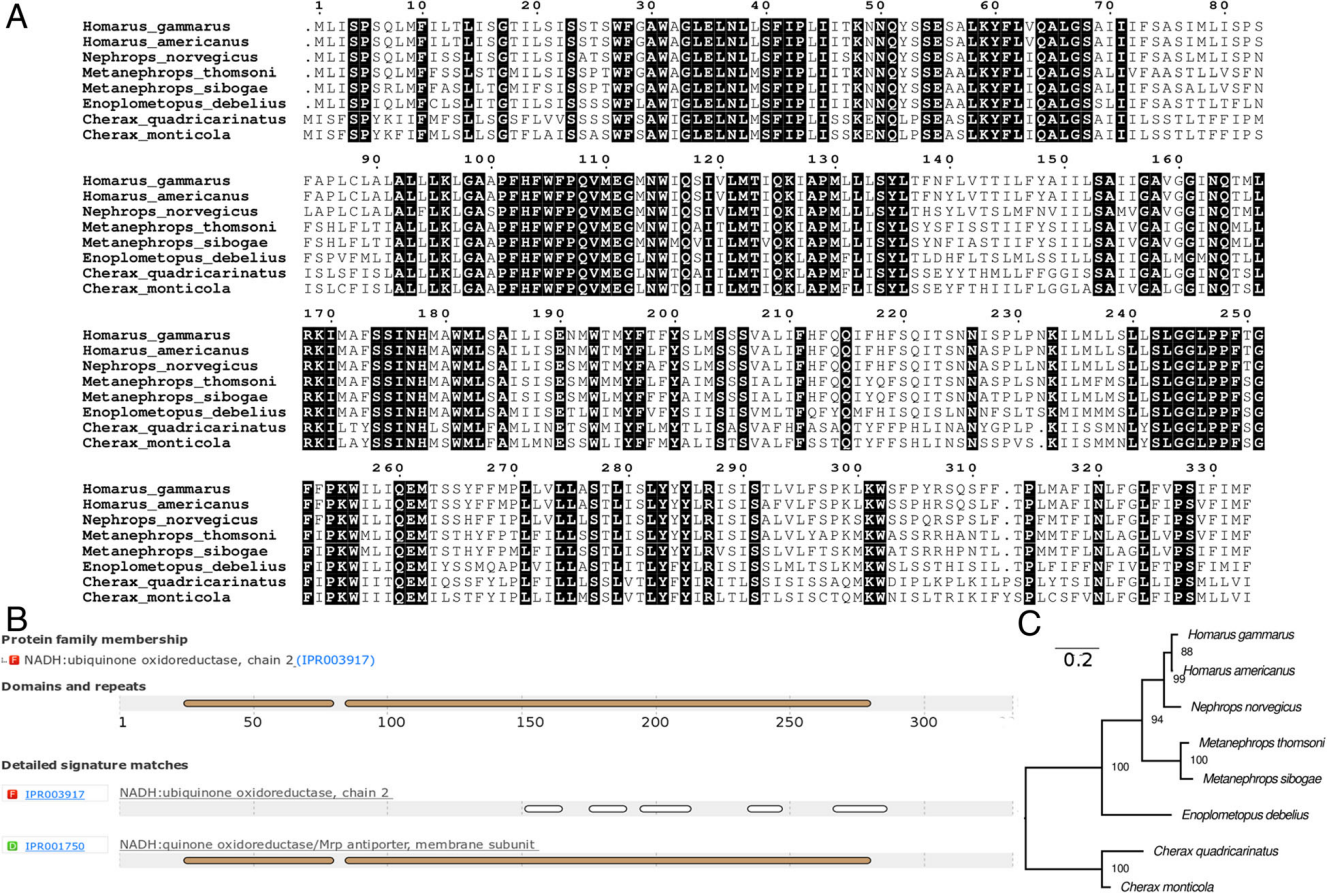
Identification and *in-silico* functional verification of the previously missing mitochondrial *nad2* in *H. gammarus*. **a** Amino acid alignment of the *H. gammarus nad2* protein with other lobsters showing high sequence conservation (residues in black boxes). Numbers above alignment indicate amino acid position. **b** Interproscan Identification of conserved protein domains in the *H. gammarus nad2* protein (**c**) Maximum likelihood tree of *nad2* proteins rooted with *Cherax* spp. as the outgroup. Node labels indicate IqTree ultra fast bootstrap support values and branch length indicates the number of substitutions per site

**Fig. 2:**
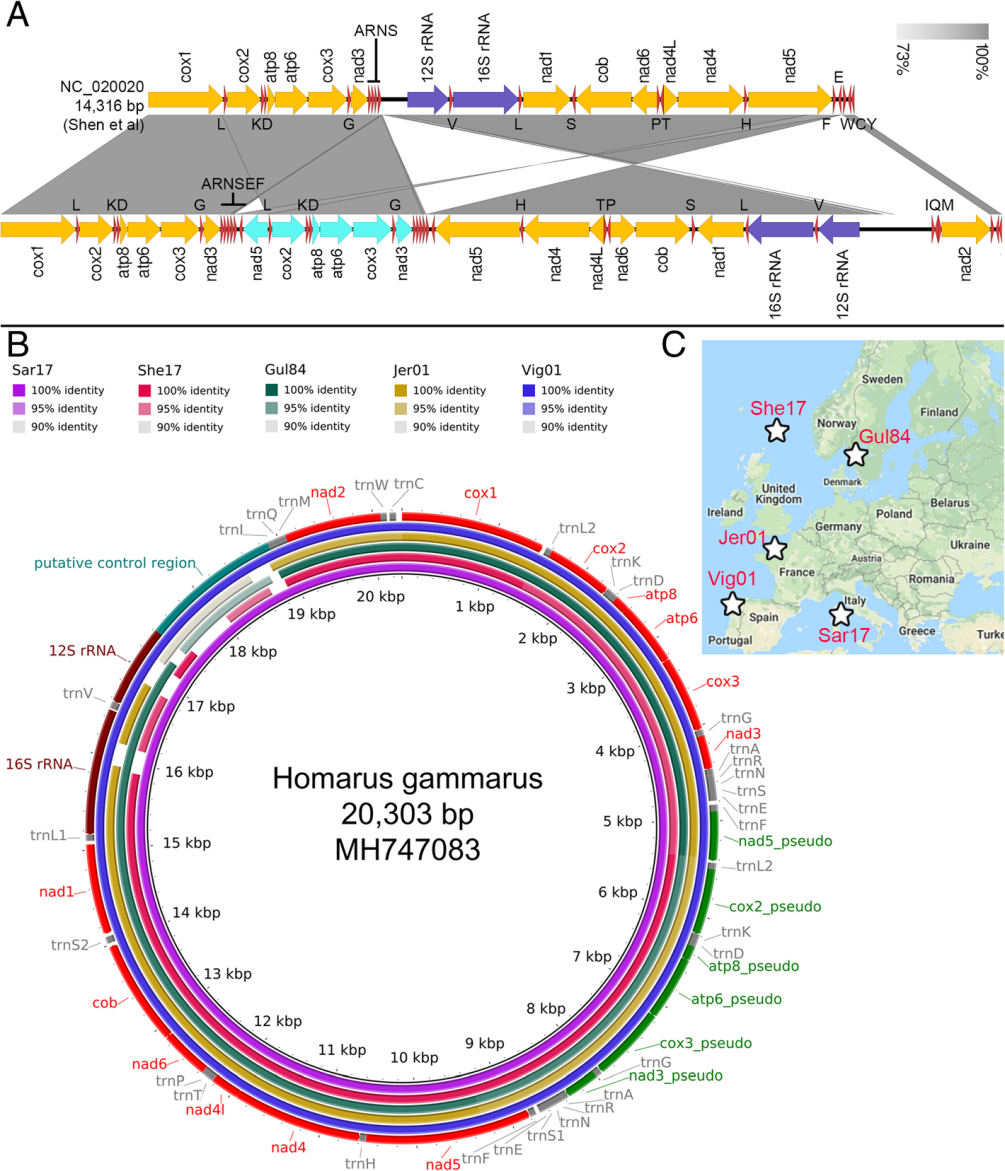
Presence of *nad2* can be detected in at least five *H. gammarus* individuals collected from various European regions. **a** Linear gene organization comparison of the previous reported *H. gammarus* mitogenome with that reported in this study confirming the absence of *nad2* in the original report in addition to substantial structural variation. The direction of arrow indicates transcription orientation. Orange, teal, purple and red arrows correspond to protein-coding genes, putative pseudogenes, rRNAs and tRNAs, respectively. **b** Circular comparison of the reconstructed mitogenomes showing significant nucleotide alignment across the entire newly assembled *H. gammarus* mitogenome with minor assembly gaps in the high-AT ribosomal rRNA regions (See Fig. 3e for details). **c** Sampling map of the five*H. gammarus* individuals with their exact location indicated by a star symbol

A total of 6,029,826 paired-end reads (~ 1.5 gigabases, SRA accession:SRP157954) were generated from MiSeq sequencing. Such a sequencing output is typically sufficient for the reconstruction of mitogenome via reference mapping or de novo assembly approach. Contrary to the previously reported loss of *nad2* in *H. gammarus* [9] (Fig. 2a), mitogenome reconstruction using *H. americanus nad2* as the bait generated a contig which spans the entire *nad2* gene of *H. gammarus* in addition to other bona fide mitochondrial genes in its vicinity with. Most contig extensions occurring at the 3′ end of the bait (Table 1), suggesting a difficult-to-assemble region possibly in the form of long repeats upstream of the *nad2* gene. The translated ORF of the assembled *nad2* shows high pairwise amino acid identity (96.7%) to that of *H. americanus* with only 11 amino acid mismatches out of 333 aligned protein length (Fig. 1a). Protein domains associated with NADH dehydrogenase subunit 2 are present in the translated ORF (Fig. 1b), suggesting a similar function. Maximum likelihood tree construction based on amino acid alignment provides phylogenetic support for its annotation as *nad2* given its well-supported monophyletic clustering (88% ultrafast bootstrap support) with the *nad2* protein of *H. americanus* (Fig. 1c).

**Table 1:** Performance of different assemblers in the recovery of *Homarus gammarus* mitogenome

Assembler	Contig Length (bait/contigID)	Protein coding gene (number of intact genes)
MITObim v. 1.9	14,885 bp (rrnL)	*nad1, cytb, nad6, nad4L, nad4, nad5, nad3, cox3, atp6, atp8, cox2, cox1, nad2* (13)
	13,143 bp (cytb)	*nad2, cox1, cox2, atp8, atp6, cox3, nad3, nad5, nad4, nad4L, nad6, cytb, nad1* (13)
	6984 bp (nad2)	*nad2, cox1, cox2, atp8, atp6, cox3, nad3, nad5, cox2* (9)
Megahit v1.1.2	5162 bp (ctg154136)	*nad2, cox1, cox2, atp8, atp6, cox3* (6)
	4045 bp (ctg252123)	*cox3*^*a*^*, atp6, atp8, cox2*^*a*^*, nad5*^*b*^*, nad3, cox3* (4)
	10,025 bp (ctg253367)	*nad1, cytb, nad6, nad4L, nad4, nad5, nad3, cox3* (8)
NOVOplasty v2.7.1	22,032 bp	*nad2, cox1, cox2, atp8, atp6, cox3, nad3, nad5, cox2*^*a*^*, atp8, atp6, cox3*^*a*^*, nad3, nad5, cox2*^*a*^*, atp8, atp6, cox3*^*a*^*, nad3, nad5, cox2*^*a*^*, atp8, atp6, cox3*^*a*^*, nad3, nad5, cox3*^*a*^*, atp6, cox3*^*a*^*, nad3* (8)

^*a*^Internal stop codon ^*b*^ Fragmented

### Illumina-based genome skimming approach fails to generate the complete *Homarus gammarus* mitogenome assembly

Both reference mapping (single-gene bait) and de novo assembly based on Illumina-only short reads failed to generate contigs that are longer than 15 kb or contain flanking identical region typically required to infer mitogenome circularity. The longest MITObim assembly was achieved using the *H. americanus cob* gene as the initial read bait with a final assembly length of 14 kb covering all 13 PCGs (Table 1). On the other hand, BlastX search against the de novo-assembled contigs identified multiple small mitochondrial contigs, some of which exhibit significant homology to one another. For example, ctg154136 (5 kb) and ctg252123 (4 kb) from the MEGAHIT assembly harbor a nearly similar set of PCGs although the some of the PCGs in the latter contig are likely pseudogenes.

### Nanopore long-read assembly confirms mitogenome circularity

An overnight run of a used nanopore flow cell generated 40,687 reads with an accumulated length of 100 megabases and N50 of 2811 bp. The basecalled fastq files were archived in Zenodo (10.5281/zenodo.1345356). Approximately 0.19% of the sequencing reads were mapped to the *H. americanus* and *H. gammarus* mitogenomes (255 kb, N50 of 4639 bp). De novo assembly of the mapped long-reads generated a 26 kb contig suggested to be “circular” by CANU. This was subsequently verified by the circular representation of the assembly graph in Bandage (Additional file 1: Figure S1A). Self-to-self nucleotide alignment of the contig indicates that up to 6 kb of its flanking regions exhibit strikingly high similarity, thus providing additional evidence for mitogenome circularity (Additional file 1: Figure S1B). Four rounds of Pilon polishing identified and corrected 402 indels/SNPs in the initial CANU-assembled contig. Subsequent polishing of the circularized and re-oriented contig corrected an additional 5 indels/SNPs, generating a final, complete and circular mitogenome with a total length of 20,303 bp and 31.68% GC-content. MITOS annotation confirmed the presence of the typical 13 PCG but also identified 6 duplicated PCGs some of which contained internal stop codons and/or frameshift mutation. The final polished and annotated mitogenome assembly has been deposited in GenBank under the accession number MH747083.

### Consistent recovery of *nad2* in *H. gammarus* individuals from different populations

Linear comparison of the recently published *H. gammarus* complete mitogenome (GenBank: NC_020020) with our new assembly revealed three substantial structural variations namely a tandem duplication involving the *cox2-atp8-atp6-nad3* protein coding genes, a large inversion and as previously highlighted, the absence of *nad2* and a few tRNAs (Fig. 2a). On average 868 Mb (670–1128 Mb) of Illumina sequencing data were generated for each of the *H. gammarus* individuals and deposited in the SRA database (Bioproject ID: PRJNA486050). Reference-based mapping assembly successfully reconstructed the near-complete mitogenome except for samples She17_01 and Jer01 that contain minor assembly gaps in the ribosomal genes and non-coding control region (Fig. 2b and Additional file 2: Data 1). Notably, significant nucleotide alignment to the *H. gammarus* mitochondrial *nad2* gene with high pairwise identity was observed across all five individuals (Fig. 2c).

### Different lines of evidence indicate partial tandem duplication in the *Homarus gammarus* mitogenome

**Fig. 3:**
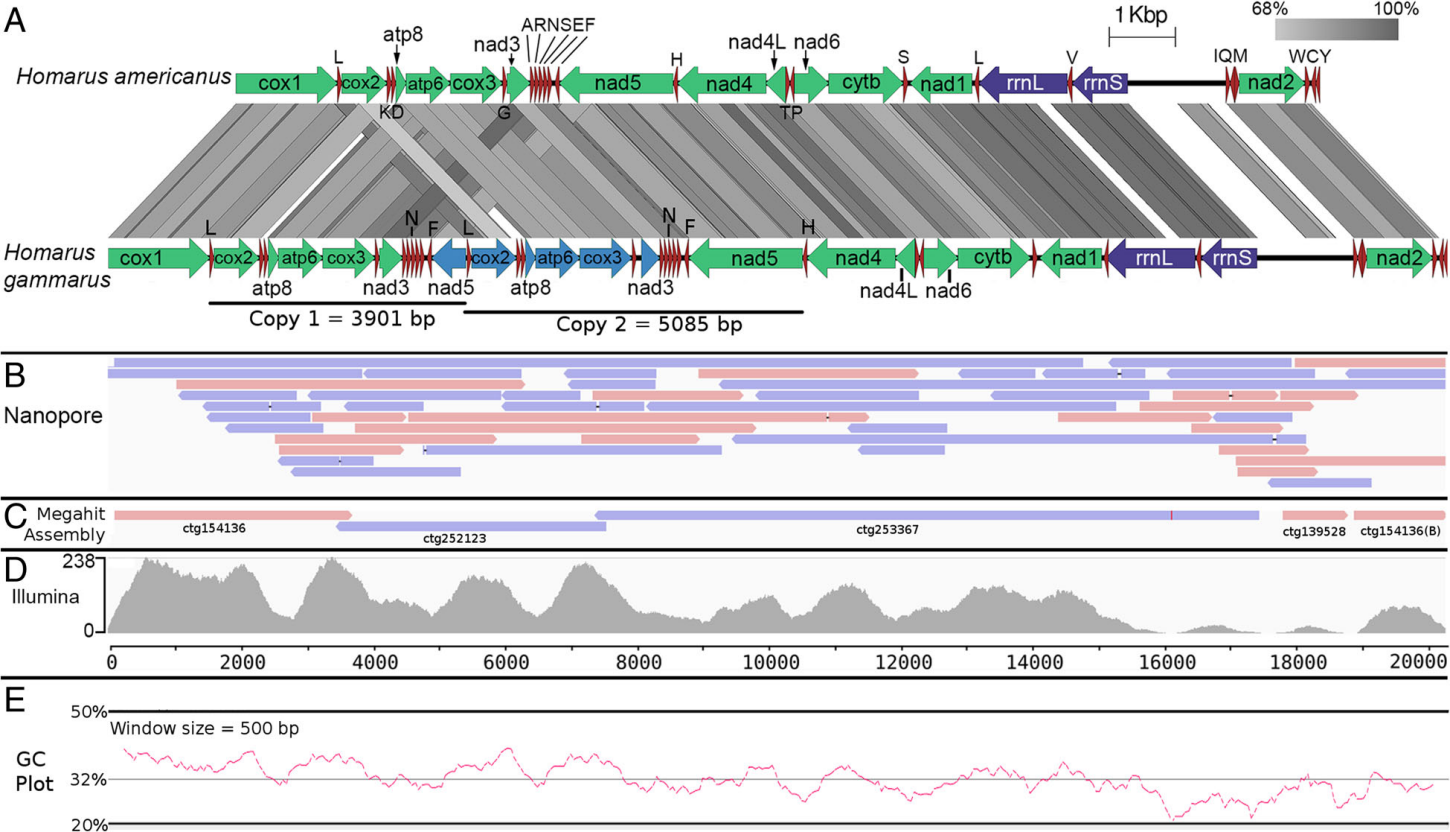
Tandem duplication in the *H. gammarus* mitogenome. **a** Linear gene organization comparison of the *H. gammarus* mitogenome with that of *H. americanus* (tblastx) showing broadly similar gene organization except for the presence of a tandemly duplicated region (*cox2-atp8-atp6-cox3-nad3-nad5*) in *H. gammarus* with dissimilar length. The direction of arrow indicates transcription orientation. Green, blue, purple and red arrows correspond to protein-coding genes, putative pseudogenes, rRNAs and tRNAs, respectively. **b** Minimap2 alignment of nanopore reads to the mitogenome showing broadly even read coverage with some extra long reads spanning the duplicated regions. **c** Minimap alignment of Megahit Illumina-only assembly indicating the presence of contigs corresponding to both duplicated regions presumably due to the presence of substantial nucleotide divergence. **d** Bowtie2 mapping of Illumina reads to the mitogenome showing uneven coverage that appears to correlate with the (**e**) positional (500 bp window size) GC-content of the mitogenome

A gene region consisting of *cox2, atp8, atp6, cox3*, *nad3,* and *nad5* as well as their neighboring tRNAs were present in two copies and in tandem in the newly assembled mitogenome. One of the *nad5 *genes was substantially shorter with its start codon and a majority of 5′ end truncated (Fig. 3a). The observed duplication was not due to sequencing or assembly error as multiple Nanopore reads spanned across both duplicated gene regions (Fig. 3b). In addition, de novo assembly using Illumina-only reads also recovered two separate contigs corresponding to each of the duplicated region suggesting that sufficient nucleotide dissimilarity exists between the repeats to prevent collapsing of contigs by the assembler into a single contig (Fig. 3c). Substantial and similar coverage of Illumina reads to both regions lends additional support to the veracity of the nanopore sequences. Interestingly, unlike the Nanopore read alignment, Illumina reads show an uneven coverage across the mitogenome that negatively correlates with the genome AT-content (Fig. 3d and e). Notably, low or near-zero read depth was observed in regions with less than 22% GC that correspond to the location of the 12S and 16S ribosomal RNA genes.

### Pseudogenization among tandemly duplicated mitochondrial genes

**Fig. 4:**
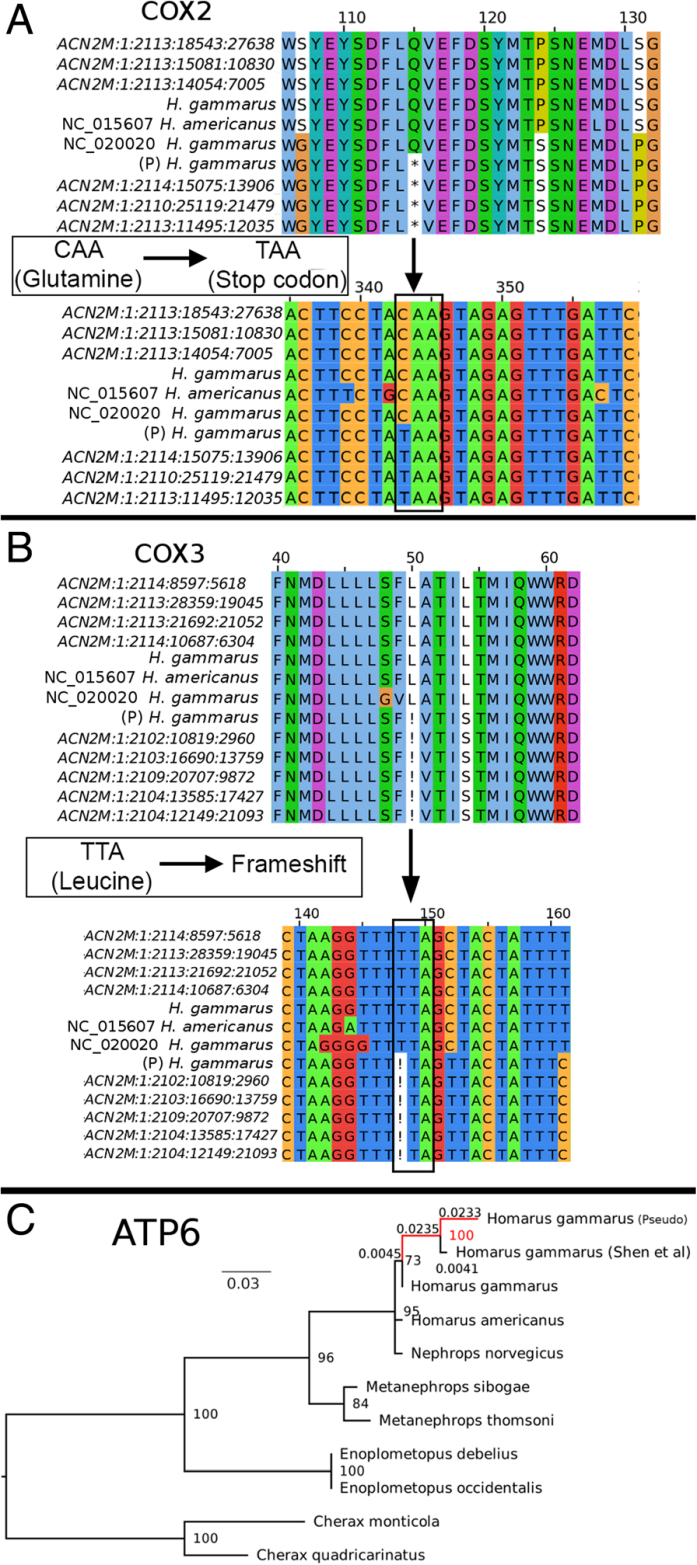
Evidence for pseudogenization among genes located in the duplicated regions. Identification of (**a**) Internal stop codon and (**b**) Frameshift mutation in one of the duplicated *cox2* and *cox3* genes, respectively, with strong support from multiple Illumina short reads. Labels with bracketed “P” indicate putative pseudogenes (**c**) Maximum likelihood tree constructed based on the protein alignment of ATP6. Labels above branches are branch lengths representing the number of amino acid substitutions per site

Duplicated genes that were disconnected from their original transcriptional track e.g. *cox2, atp8, atp6, cox3,* and *nad3* in the second duplicated region and *nad5* in the first duplicated region, appear to show signs of pseudogenization. For example, an internal stop codon was located in the duplicated *cox2* gene (Fig. 4a) while frameshift mutations were found in the *cox3*, *nad3,* and *atp8* genes (Fig. 4b). DNA mutations leading to pseudogenization were supported by multiple Illumina reads, precluding the possibility of assembly or sequencing errors. Although both duplicated atp6 genes are still intact and can be translated into protein sequence of the same length, protein similarity search using the ATPase6 of *H. americanus* as the query against both ATPase6 proteins indicates that the ATPase6 encoded by the gene located in the first duplicated region is likely the functional one as evidenced by its strikingly high protein identity to that of *H. americanus* (99.1%) compared to its duplicated partner (94.6%). Furthermore, the pseudo ATPase6 exhibits a substantially longer branch length compared to its homologs in the maximum likelihood tree (Fig. 4c), suggesting higher substitution rate presumably due to the lack of evolutionary constraints. It is also interesting to note that the pseudo ATPase6 clustered with the previously published *Homarus gammarus* ATPase6 that also shows a similar branch length pattern.

## Discussion

The veracity of the current European lobster reference mitogenome in the NCBI database (Accession Number: NC_020020.1) is questioned given its unusual features e.g. missing *nad2*, large mitogenome inversion and translocated tRNAs [9, 10, 24] that were not observed in its close relative, the American lobster, *Homarus americanus* [8]. Although subsequent PCR and Sanger sequencing has been carried out on additional individuals to validate the observed abnormalities [10], a secondary validation by a separate group using different sequencing technology is highly desirable to provide confirmation of this atypical crustacean mitogenome sequence. We, therefore, sequenced another individual of *Homarus gammarus* using Illumina technology, which removes the need for the laborious and presumably error-prone steps required in traditional mitogenome sequencing such as long-range PCR and primer walking that may lead to the unintentional misassembly of mitogenome.

The absence of a repetitive region and/or duplicated gene region in the previously reported *H. gammarus* mitogenome [9] (Fig. 2a) suggests that it should be possible to recover a complete mitogenome using Illumina short reads as demonstrated in various crustacean mitogenome reports [16, 21, 22, 56, 57]. However, none of our attempted Illumina-only assemblies recovered a circular mitogenome, indicating the potential presence of a problematic region in the mitogenome that could not be resolved, presumably due to the limitation of short reads in recovering regions with long repeats. Only by leveraging on the availability of Oxford Nanopore long-read technology, were we able to confidently close this mitogenome. In doing so, we uncovered the presence of an unforeseen duplicated region in the *H. gammarus* mitogenome in addition to the presence of the *nad2* gene which was previously reported as missing [9]. The identification of multiple Nanopore reads spanning the entire duplicated region lends support to the authenticity of the duplication observed in the mitogenome assembly. Furthermore, by employing the Nanopore PCR-free approach to sequencing e.g. by directly sequencing the native DNA, we can safely disregard misassembly due to chimeric sequences from PCR-amplified products commonly associated with long-range PCR [58, 59]. It is also fortuitous that the duplicated regions have accumulated sufficient mutation resulting in the assembly of both regions as separate contigs using Illumina-only reads which provides additional and complementary support for the presence of tandem duplication in the *H. gammarus* mitogenome recovered from the Nanopore reads. The length of the observed large inversion between Shen et al [9] *H. gammarus* assembly (GenBank: NC_020020) and ours (GenBank: MH747083) (Fig. 2a) is consistent with the estimated amplicon length generated by the primers used for long-range PCR raising the possibility that misassembly could occur during the scaffolding of the assembled amplicons.

The clustering of the published *H. gammarus* AT6ase with the pseudo-ATP6ase recovered in this study coupled with its notably longer branch length compared to the bona fide *H. gammarus, H. americanus,* and *Nephrops norvegicus* ATP6ases strongly suggests that this *atp6* gene is a chimera of the bona fide and pseudogenes. The use of long-range PCR can result in the amplification of both duplicated region that will be considered as a single PCR product given their near-identical amplicon size. Subsequent Sanger sequencing of the “mixed” amplicons will result in the presence of non-random mixed peaks in the sequencing chromatogram that will likely be haphazardly regarded as heteroplasmy. Depending on the peak intensity, alleles originating from the other duplicated region may be called leading to the assembly of a chimeric sequence.

Based on an initial analysis of 100 *H. gammarus* individuals, Triantafyllidis et al. used restriction fragment length polymorphism (RFLP) profiling to show that a 3 kb mitochondrial region (amplified using the Hom3F and Hom3R primers) containing *cox2, cox3, atp6, atp8, nad3* and several tRNAs is the most variable region and hence selected that region for a population genetic study of 4018 individuals collected throughout the geographical range of the species [7]. This is quite surprising given that the 3 kb region does not contain the control region which is known to be the most polymorphic region in mitochondrial DNA [60], suggesting an unknown source of nucleotide variation in addition to population-specific genetic variation. Similarity search of Hom3F and Hom3R primers against the newly assembled *Homarus gammarus* mitogenome indicates that primer binding sites are present in both of the duplicated regions which will lead to the amplification of two amplicons with near exact length (~ 2.8 kb) (Additional file 3: Table S1), and may well have confounded the results of Triantafyllidis et al. [7].

Mapping of Illumina reads to the mitogenome revealed a GC-dependent coverage variation that was not observed in the Nanopore read alignment. Since the standard Nanopore library preparation does not involve any PCR step, the uneven mapping coverage is most definitely caused by PCR amplification bias during Illumina library preparation which is known to under-represent AT-rich fragments [61, 62]. AT-rich regions are prevalent in the control region and ribosomal RNA genes. Such mitogenome regions will be harder to assemble due to low sequencing depth, leading to gaps in the mitogenome assembly and the incorrect inference of mitogenome linearity. Although our study highlights the value of Nanopore long-read technology in resolving a difficult-to-assemble mitogenome, we are conservative in advocating its use in routine mitogenome sequencing due to its stringent requirement for high-quality and intact gDNA. Having said that, the obvious advantage that Nanopore long reads offer in spanning repetitive sequencing make it a powerful new tool for elucidating or confirming duplicated mitochondrial genes and difficult-to-assemble mitogenomes in animals. Another third generations sequencing approach that can be used to improve a mitogenome assembly is PacBio technology albeit its reliance on polymerase chain reaction to generate sequencing data may reduce its efficiency in the sequencing of problematic region, as encountered in Illumina sequencing in this study.

Lastly, we emphasize as reported in other scientific domains, that the absence of evidence (for a mitochondrial gene) is not necessary evidence of absence. A gene may fail to be recovered due to limitation in a wet lab protocol or bioinformatics pipeline as we demonstrated for the *nad2* gene in *H. gammarus* in this study and others have demonstrated for the *atp8* gene in bivalve mollusks [63]. Thus, researchers need to be skeptical of assertions that a mitochondrial gene is missing and make efforts to verify that there really is a genuine evidence of absence.

## Conclusions

We demonstrate the utility of Oxford Nanopore long-read in resolving problematic mitogenome assemblies by re-sequencing the mitogenome of the European lobster that was previously reported to exhibit several atypical features. In addition to showing substantial deviation from the previously published mitogenome, we identified new gene features that were missed by previous studies including the complete recovery of the *nad2* gene and a large tandem duplication consisting of six protein-coding genes with evidence of pseudogenization. The power of long reads in resolving repetitive regions and accurately identifying tandemly duplicated mitochondrial gene regions highlights the value of new sequencing tools for evolutionary biologists to confidently infer novel mitochondrial gene organization. Furthermore, the veracity of unusual mitochondrial gene rearrangements recovered using first and second sequencing technologies, particularly those used to make phylogenetic inferences of evolutionary scenarios, can now be effectively tested using Oxford Nanopore long reads, marking another step forward in mitogenomics.

## Additional files

Additional file 1: **Figure S1.** Long-read only assembly enables the complete assembly of *H. gammarus* mitogenome. (A) Bandage visualization of the CANU assembly graph corresponding to the mitogenome contig. (B) Self-against-self comparison of the mitogenome contig in EasyFig with up to 6 kb of flanking regions exhibiting high nucleotide similarity (~ 98%) (TIF 76 kb)

Additional file 2: **Data 1.** Mitogenome assemblies in fasta format of 5 additional *Homarus gammarus* sampled from distant location in Europe (See Fig. 2c for details) (TXT 99 kb)

Additional file 3: **Table S1.** Binding sites of I_Hom3F and I_Hom3R primers on the newly assembled Homarus gammarus mitogenome and the estimated PCR product sizes. (PDF 16 kb)

## Abbreviations

PCG: Protein Coding Gene
PCR: Polymerase Chain Reaction
SDS: Sodium Dodecyl Sulfate
SNP: Single Nucleotide Polymorphism
