# Supplementary figures and images for: Absence of evidence is not evidence of absence: Nanopore sequencing and complete assembly of the European lobster (Homarus gammarus) mitogenome uncovers the missing nad2 and a new major gene cluster duplication
Source: BMC Genomics. 2019 May 3;20:335. doi: 10.1186/s12864-019-5704-3 (PMC6500004; doi:10.1186/s12864-019-5704-3)

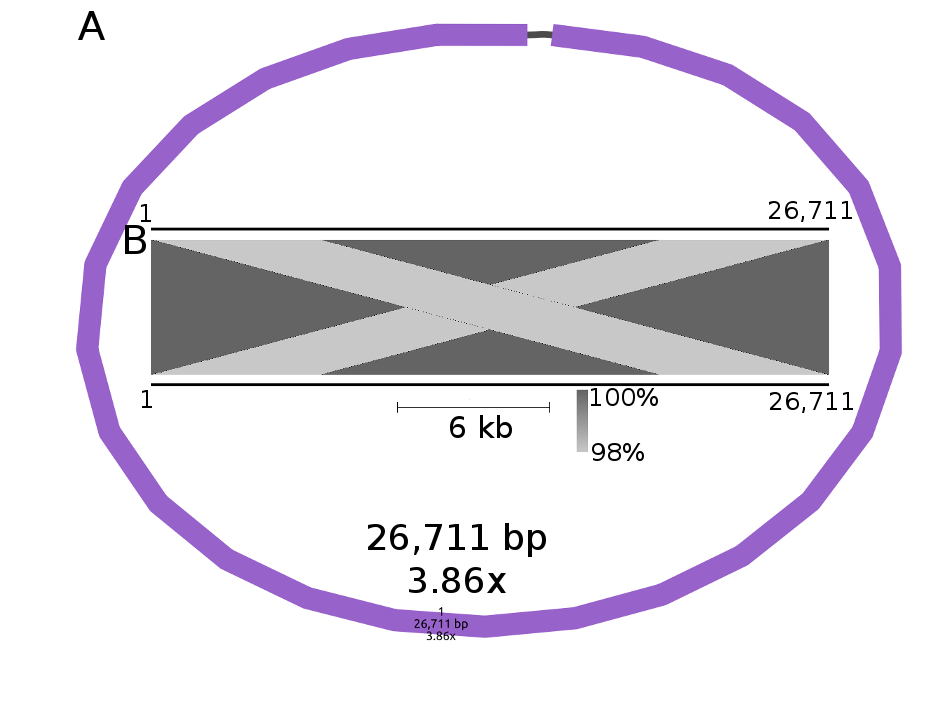

Supplement: Supplementary file 1 — Figure S1. Long-read only assembly enables the complete assembly of H. gammarus mitogenome. (A) Bandage visualization of the CANU assembly graph corresponding to the mitogenome contig. (B) Self-against-self comparison of the mitogenome contig in EasyFig with up to 6 kb of flanking regions exhibiting high nucleotide similarity (~ 98%) (TIF 76 kb) [file 12864_2019_5704_MOESM1_ESM.tif]
