# Supplementary material for: Absence of evidence is not evidence of absence: Nanopore sequencing and complete assembly of the European lobster (Homarus gammarus) mitogenome uncovers the missing nad2 and a new major gene cluster duplication
Source: BMC Genomics. 2019 May 3;20:335. doi: 10.1186/s12864-019-5704-3 (PMC6500004; doi:10.1186/s12864-019-5704-3)
Supplement: Supplementary file 3 — Table S1. Binding sites of I_Hom3F and I_Hom3R primers on the newly assembled Homarus gammarus mitogenome and the estimated PCR product sizes. (PDF 16 kb) [file 12864_2019_5704_MOESM3_ESM.pdf]

Primer_binding

Query (Primer)	Subject(mitogenome)	Identity	AlignmentLength	qstart	qend	sstart	send	evalue	bitscore	Est. Size
I_Hom3F_3kb	Hgam	100	18	3	20	1730	1747	8.28E-06	34.4	2836
I_Hom3R_3kb	Hgam	95.652	23	1	23	4566	4544	8.54E-07	38.1	
I_Hom3F_3kb	Hgam	95	20	1	20	5630	5648	0.00011	30.7	2835
I_Hom3R_3kb	Hgam	95.652	23	1	23	8465	8443	8.54E-07	38.1	
